# Access to primary healthcare Services in Conflict-Affected Fragile States: a subnational descriptive analysis of educational and wealth disparities in Cameroon, Democratic Republic of Congo, Mali, and Nigeria

**DOI:** 10.1186/s12939-021-01595-z

**Published:** 2021-12-11

**Authors:** Marwa Ramadan, Hannah Tappis, Manuela Villar Uribe, William Brieger

**Affiliations:** 1 Department of International Health, Johns Hopkins Bloomberg School of Public Health, Baltimore, MD USA; 2 Technical Leadership and Innovations Office, Jhpiego, Baltimore, MD USA; 3 Health Nutrition and Population Global Practice, World Bank Group, Washington, DC USA

**Keywords:** Accessibility of health services, Armed conflict, Fragile states, Healthcare disparities, Primary healthcare, Central Africa, Western Africa

## Abstract

**Background:**

Measuring and improving equitable access to care is a necessity to achieve universal health coverage. Pre-pandemic estimates showed that most conflict-affected and fragile situations were off-track to meet the Sustainable Development Goals on health and equity by 2030. Yet, there is a paucity of studies examining health inequalities in these settings. This study addresses the literature gap by applying a conflict intensity lens to the analysis of disparities in access to essential Primary Health Care (PHC) services in four conflict-affected fragile states: Cameroon, Democratic Republic of Congo, Mali and Nigeria.

**Methods:**

For each studied country, disparities in geographic and financial access to care were compared across education and wealth strata in areas with differing levels of conflict intensity. The Demographic Health Survey (DHS) and the Uppsala Conflict Data Program were the main sources of information on access to PHC and conflict events, respectively. To define conflict intensity, household clusters were linked to conflict events within a 50-km distance. A cut-off of more than two conflict-related deaths per 100,000 population was used to differentiate medium or high intensity conflict from no or low intensity conflict. We utilized three measures to assess inequalities: an absolute difference, a concentration index, and a multivariate logistic regression coefficient. Each disparity measure was compared based on the intensity of conflict the year the DHS data was collected.

**Results:**

We found that PHC access varied across subnational regions in the four countries studied; with more prevalent financial than geographic barriers to care. The magnitude of both educational and wealth disparities in access to care was higher with geographic proximity to medium or high intensity conflict. A higher magnitude of wealth rather than educational disparities was also likely to be observed in the four studied contexts. Meanwhile, only Nigeria showed statistically significant interaction between conflict intensity and educational disparities in access to care.

**Conclusion:**

Both educational and wealth disparities in access to PHC services can be exacerbated by geographic proximity to organized violence. This paper provides additional evidence that, despite limitations, household surveys can contribute to healthcare assessment in conflict-affected and fragile settings.

**Supplementary Information:**

The online version contains supplementary material available at 10.1186/s12939-021-01595-z.

## Background

Access to care is a fundamental measure of health system performance [1]. In the era of sustainable development goals (SDGs), measuring and improving equitable access to care is a necessity to achieve universal health coverage [2]. Pre-pandemic estimates showed that the majority of conflict-affected and fragile situations were off-track to meet the SDGs on health and equity by 2030 [3]. Yet, there is a paucity of studies examining health inequalities in these settings [4].

Most assessments of access to care focus first and foremost on access to primary health care. In 1978, the Alma-Ata declaration recognized the importance of access to a holistic Primary Health Care (PHC) system for improved effectiveness and responsiveness of health systems [5]. The declaration defined PHC as “essential healthcare based on practical, scientifically sound, and socially acceptable methods and technology made universally accessible to individuals and families in the community through their full participation and at a cost that the community and the country can afford to maintain at every stage of their development in the spirit of self-reliance and self-determination” [6].

Access to care can be examined from both the supply and the demand side perspectives. Upstream supply or system capacity factors such as basic equipment, infrastructure, workforce, or health financing are important to ensure the availability of services. However, health outcomes are less likely to be improved if patients face barriers to care even if high-quality services were offered at the facility [7]. Therefore, examining access based on patients’ experience and perceived barriers to care is a critical factor for achieving effective service coverage [8].

Several frameworks have been used to explain the individual use and access to health services. One of the earliest and the most common was the socio-behavioral model developed by Ronald Andersen [9]. In his model, access to care was presented as the outcome of three types of factors: predisposing factors such as demographics and personal characteristics; enabling factors such as health insurance, personal and community resources; and health needs such as degree of illness or health status.

The Primary Healthcare Performance Initiative (PHCPI) emphasized the importance of measuring PHC access based on patients’ perceived barriers to care. PHCPI is an initiative launched in 2015 by the Bill & Melinda Gates Foundation, the WHO, the United Nations Children’s Emergency Fund, and the World Bank Group, in collaboration with Ariadne Labs and Results for Development to narrow the measurement gap in PHC service delivery [10]. In the PHCPI’s measurement framework, access to PHC services is examined from the patients’ perspective and is defined as the absence of both the geographic and financial barriers to care [10]. Financial barriers to care mainly refer to the cost of receiving care, including out-of-pocket payment, user fees, and transportation fees, while geographic barriers refer to physical challenges as distance to health facilities and availability of transportation. Although a number of PHCPI supported countries are affected by conflict and fragility, the impact of these conditions was not considered in the initiative’s initial strategy and measurement framework development. This may be, in part, because the literature on measurement of primary health care performance in conflict-affected and fragile situations is sparse.

Previous studies have examined health disparities in relation to conflict by comparing disparities between rather than within countries [4]. Others have shown how conflict negatively impacts the social, political, and economic institutions within a state resulting in a vicious circle of conflict and fragility. The health sector, in particular, can suffer grave consequences both directly and indirectly, threatening access to essential services [11–13]. Furthermore**,** lack of adequate access to essential health services may in-turn, augment a sense of insecurity within a community [14, 15]. However, the extent to which conflict affects health services distribution among various social strata within a state is yet to be investigated. This study addresses the literature gap by applying a conflict intensity lens to the analysis of disparities in access to essential PHC services within conflict-affected fragile states.

## Materials and methods

In this study, we examined disparities in PHC access in four conflict-affected fragile states. We defined PHC access as the absence of both the geographic and financial barriers to care, based on the PHCPI measurement framework and vital signs profile [10]. For each studied country, disparities in geographic and financial access to care were compared across education and wealth strata in areas with differing levels of conflict intensity; specifically, access disparities reported by women living in neighborhoods (household clusters) with medium or high-intensity conflict were compared to access disparities reported by women living in neighborhoods with no or low-intensity conflict. To define conflict intensity at the household cluster level, organized violence events and their associated fatalities were geographically linked to DHS household clusters’ location. Three measures of disparities were computed, and each was compared based on the intensity of conflict the year the DHS data was collected (Cameroon 2018, DRC 2013, Mali 2018, Nigeria 2018).

### Selection of the studied contexts

In 2020, the World Bank identified 39 fragile and conflict-affected situations [16]. To systematically select countries for this study, we applied the following inclusion criteria to the specified list: 1) the country had a DHS survey in the past ten years (2010-2020) to ensure the availability of data on access to PHC using standardized indicators; 2) there was an ongoing armed conflict the year the household survey was conducted, with data on violent events and conflict-related deaths available in the UPPSALA Conflict Data Program (UCDP) database. Armed conflict was defined as the presence of at least 25 conflict-related deaths per year [17]; and 3) the geographic locations of DHS household clusters were publicly available to allow for spatial analysis, including linkage with armed conflict location data. Applying these criteria yielded five countries: Cameroon, the Democratic Republic of Congo (DRC), Mali, Myanmar, and Nigeria. Since this study does not aim for geographic comprehensiveness, but rather the detection of common patterns of disparities across conflict-affected fragile states, we excluded Myanmar from the analysis as it was the only country belonging to a geographically different context.

### Data sources

The DHS was used as the source of information on the geographic and financial barriers to care as recommended by the PHCPI methodology [18]. DHS is a nationally representative household survey and a vital source of information on population, health, and nutrition indicators in more than 90 countries [19]. The standard DHS survey is conducted every five years using a large sample size (5000-30,000 households per survey). Based on the availability of census information, most countries apply a two-stage stratified sampling technique. The first stage includes selecting enumeration areas (EA) or clusters with a probability proportional to EA size. An equal probability systematic sampling strategy is then applied in the second stage to draw a fixed number of households per cluster. Each survey can comprise various research tools, including multi-module questionnaires, geographic information collection, and occasional biomarkers collection. The survey duration typically ranges between 18 and 20 months [19]. In the four studied contexts, a two-stage stratified sample was conducted except for in new provinces [20] and some parts of established provinces in DRC, where a three-stage sample was used. Table S1 provides more details on the characteristics of the demographic health surveys included in the analysis (see Additional file 1).

Conflict data were obtained using the UCDP database [17, 21]. UCDP is the primary global source for data on armed conflict and organized violence. UCDP’s definition of armed conflict became the international standard allowing for systematic analysis of temporal trends and cross-country comparisons. The unit of the analysis in the UCDP database is an ‘event’ - an instance of fatal organized violence defined as: “The incidence of the use of armed force by an organized actor against another organized actor, or against civilians, resulting in at least one direct death in either the best, low or high estimate categories at a specific location and for a specific temporal duration.” [17]. Each event meeting the former criteria is recorded as one line in the database. Events with uncertain information on the number of fatalities or those with no reported deaths are excluded [17, 21].. In this study, we extracted and analysed all events satisfying the UCDP definition of an event of organized violence.

### Metrics

#### Access metrics

We analysed both geographic and financial access to PHC services using the DHS question on perceived barriers to care by interviewed women [19]. Perceived barriers due to distance were defined as the percentage of women aged (15-49) years who report specific problems in accessing care when they are sick due to the distance travelled for treatment. Similarly, perceived barriers due to treatment costs were defined as the percentage of women aged (15-49) years who report specific problems in accessing care when they are sick due to issues related to getting money for treatment. A PHC access index score was then computed based on the PHCPI methodology [22] as the average of not perceiving geographic barriers and financial barriers to care as follows:

$$\boldsymbol{Access}\kern0.5em \boldsymbol{index}\ \boldsymbol{score}=\frac{\left(\mathbf{100}-\%\boldsymbol{women}\ \boldsymbol{with}\ \boldsymbol{geographic}\ \boldsymbol{barriers}\right)+\left(\mathbf{100}-\%\kern0.5em \boldsymbol{women}\ \boldsymbol{with}\ \boldsymbol{financial}\ \boldsymbol{barriers}\right)\ }{\mathbf{2}}$$

#### Disparities metrics

We used the DHS educational status and wealth index variables to define the educational and wealth disparities respectively:

The DHS defines an educational status variable (v106) as the highest level of education attended but not necessarily completed. It is further subdivided into the following categories: no education, primary, secondary, and higher than secondary [23]. Such classification may vary by country, but the standard classification has been consistently reported in the four-studied countries. In this analysis, we define educational disparity as the difference in access to PHC services among women with varying education levels.

The DHS wealth index (v190) is a composite measure that gives a general idea of living standards based on household access to water and sanitation, ownership of certain assets such as TV, bicycles, and household construction material. The index is calculated at the household level using a standardized score for each asset. The individuals are then ranked based on the household’s total score and divided into five population wealth quintiles: lowest, second, middle, fourth, and highest [23]. In this analysis, we define economic or wealth disparity as the difference in access to PHC services comparing women with varying wealth quintiles.

### Data analysis

We geographically and temporally linked household clusters to organized violence events located within a 50-km distance from the centroid representing the cluster location. The size of the buffer zone was decided based on previous studies examining the effect of armed conflict on maternal and child health outcomes [24, 25]. Conflict intensity was defined as a binomial variable with “medium or high” conflict intensity = 1 and “no or low” conflict intensity = 0. A cut-off of more than two conflict-related deaths per 100,000 population (according to the total number of fatalities best estimate) per household cluster population was used to define medium or high-intensity conflict. This cut-off point was selected based on the World Bank definition of low-intensity conflict [26]. The total population size per cluster, according to 2015 estimates, was used as a reference point for classifying conflict exposure in each studied context. The 2015 estimates were selected as they were the closest estimates of cluster size in the DHS environmental database in the four studied countries. In Nigeria, 13 clusters in Borno state and one cluster in Yobe state were excluded from the analysis due to the lack of information on the total population size. Additionally, we excluded one cluster in the extreme north region in Cameroon with available environmental covariates but no corresponding DHS health variables.

For each health indicator, three measures of disparities were computed: an absolute measure of inequality, a concentration index, and a multivariate logistic regression coefficient. Several studies recommended the use of multiple measures while addressing health disparities [27–33]. For example, Sully et al. [31] highlighted the importance of incorporating relative, absolute, and population impact measures to understand inequalities. Similarly, Alonge et al. [32], in their review of the utility and limitations of disparity measures, concluded that there is no perfect measure of disparity, and each quantifies some aspect of health disparity. They also highlighted the importance of combining measures for a more comprehensive evaluation of health programs. The same conclusion was reached by Houweling et al. [33], who recommended combining both the relative and absolute measures of inequalities while considering the overall level of the outcome.

In this analysis, we also viewed the three measures of disparities as complementary rather than alternatives, each contributing to one aspect of disparity understanding. For instance, the absolutes difference would help estimate and interpret the magnitude of disparity between the highest and the lowest sub-groups; however, it would not consider the indicator’s distribution across all other sub-groups. The latter was covered by the concentration index, which assessed relative inequalities and also allowed for the statistical comparison of inequalities between different conflict intensities. Similarly, regression coefficients carried the additional advantage of comparing groups while adjusting for other sociodemographic variables.

In this study, the absolute difference was calculated as a difference in the frequency between the highest and the lowest sub-categories of women’s educational status (secondary or more education vs. no education) and the wealth index (q5 vs. q1). The latter has also been reported as part of the PHCPI recommended indicators for measuring economic disparities in financial barriers to PHC services [10].

In contrast to the absolute difference, the concentration index with erreygers correction considered all sub-groups to analyse disparities [34]. Concentration index values range from − 1 to + 1, with positive values indicating the health metric’s concentration among the advantaged groups. Negative values indicate the concentration of the health metric among the disadvantaged groups. The closer the value is to zero, the more likely the indicator is equally distributed across all sub-groups. Previously, concentration index was mainly used to assess economic disparities as an extension of the Lorenz curve and Gini coefficient. However, an adapted version of the concentration curve and index allowed the assessment of inequality in binomial health outcomes over the distribution of other ordered categorical variables as educational groups or wealth quintiles [34]. Z statistics were used to test the difference in concentration index estimates between the two categories of conflict intensity.

Given the hierarchical nature of DHS data, which violates the assumption of observations’ independence, we performed a multilevel modeling analysis of disparities to appropriately account for additional sociodemographic variables that may affect the overall wealth or educational disparity levels. A two-level mixed-effects logit (random intercept) regression model was fitted for each studied indicator using STATA 16 “svy: melogit command”. The fitted model included the following variables: age of women in years, disparity variable (income quintile of the household or educational status of women), employment status of women (categorical), the number of children per woman (continuous), urban/rural status of the household (categorical). The model was fitted separately for the two categories of conflict intensity; then a combined model was used to test for the interaction between conflict intensity and disparity variable. Table S2 providers more details on model equations (see Additional file 1).

## Results

The total analyzed sample included 85,374 women living in four fragile countries affected by a total of 297,873 organized violence events and 1,702,818 associated fatalities in the year DHS data was collected (Cameroon 2018, DRC 2013, Mali 2018, Nigeria 2018). At the country level, the highest average intensity of conflict surrounding household clusters (within 50 km distance) was recorded in Cameroon, with an average of 465 conflict-related deaths per 100,000 population. Sub-nationally, the highest average conflict intensity per household cluster was recorded in North-west and Littoral regions in Cameroon (3051 deaths per 100,000 population and 2692 deaths per 100,000 population respectively), and North Kivu province in DRC (1041 deaths per 100,000 population). Table S3 provides more detailed description of the sociodemographic characteristics of the studied sample by conflict intensity in each of the studied contexts (see Additional file 1).

### Sub-national variation in access to PHC services

**Fig. 1:**
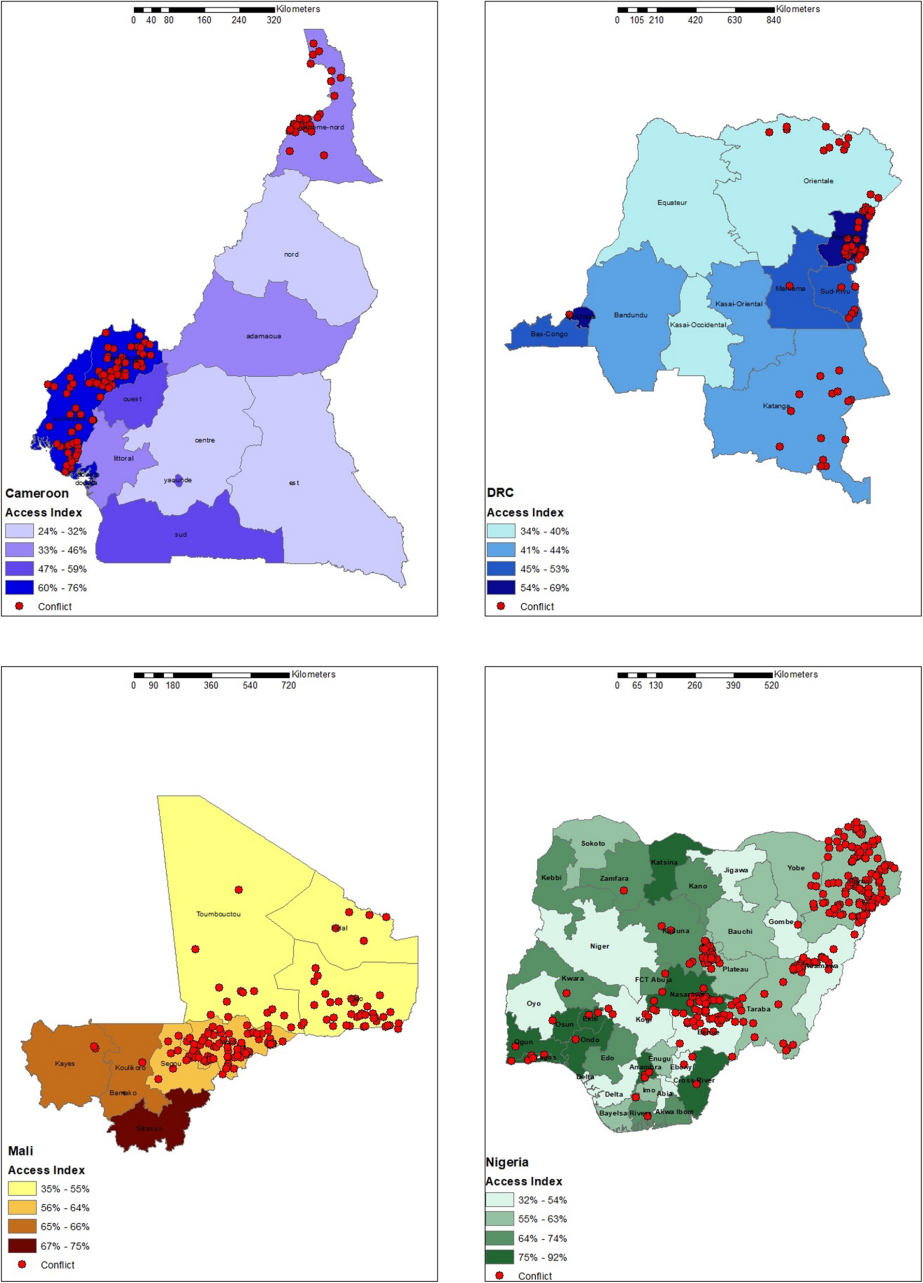
Access index score and conflict locations at the sub-national level in Cameroon, DRC, Mali, and Nigeria

In the four studied contexts, subnational variation in PHC access was observed at the state level, with the highest being in Nigeria, where the access index ranged from 32% in the Oyo state to 92% in Nasarawa, Ondo, and Osun states. In Cameroon, the PHC access index ranged from 24% in the East state to 76% in the South-west state, while in DRC, Equateur state had the lowest PHC access index of 34%, and the capital Kinshasa had the highest PHC access index of 69%. A similar sub-national variation was observed in Mali, where women living in Kidal had a 35% perceived access to PHC services compared to a PHC access index of 88% among the women living in the capital Bamako. In the four countries, variation in PHC access index score could not be visually linked to the locations of conflict events reported in the year the DHS data were collected [Fig. 1], so analysis at the household cluster level was done.

**Fig. 2:**
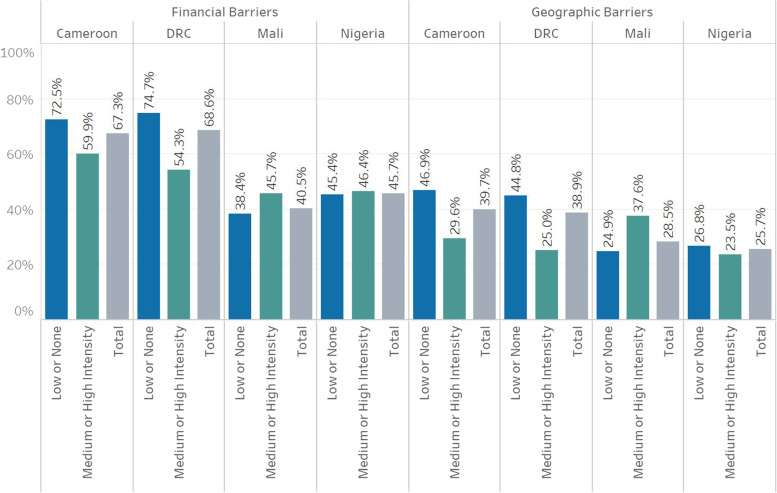
Geographic and financial barriers to PHC services by conflict intensity in Cameroon, DRC, Mali, and Nigeria

In the four studied contexts, more financial than geographic barriers to care were observed, regardless of conflict intensity. Surprisingly, household clusters surrounded by no or low-intensity conflict in Cameroon and DRC had relatively higher perceived geographic and financial barriers to PHC services compared to those surrounded by medium or high intensity conflict. In Nigeria, barriers to care were similar in both types of clusters, while Malian women living in clusters surrounded by no or low-intensity conflict had lower perceived barriers to care than those surrounded by medium or high-intensity conflict [Fig. 2].

### Disparities in geographic and financial barriers to care at the national level

In the four studied contexts, both educational and wealth disparities in access to PHC services were observed regardless of conflict intensity. Cameroonian, Congolese, Malian, and Nigerian women with no education had more geographic and financial barriers to care than women with secondary or more education. Similarly, women in the first wealth quintile in the four studied contexts had more geographic and financial barriers to PHC services than women in the fifth wealth quintile.

Educational and wealth disparities in access to PHC services were also observed when all sub-groups of educational status and wealth quintiles were considered using a concentration index. In the four studied contexts, there was a significant concentration of geographic and financial barriers among the least educated and the least wealthy regardless of the intensity of conflict surrounding their neighbourhoods. For example, in Cameroon, educational concentration indexes of − 0.25 (*p* = 0.001) and of − 0.23 (*p* < 0.001) were observed in the geographic and financial barriers to care, respectively. Similarly, wealth concentration indexes of − 0.32 (p < 0.001) and of − 0.28 (p < 0.001) were observed in the geographic and financial barriers to care.

Statistically significant relative educational and wealth disparities in PHC access were also observed after adjustment for additional sociodemographic variables using mixed-effects logistic regression. For instance, Nigerian women with secondary or more education had 39% lower odds of perceiving geographic barriers and 49% lower odds of perceiving financial barriers to access when compared with women with no education, after adjustment for additional sociodemographic variables and educational groups. Similarly, Nigerian women in the highest wealth quintile had 65% lower odds of perceiving geographic barriers and 92% lower odds of perceiving financial barriers when compared with women in the lowest wealth quintile adjusting for additional sociodemographic variables and wealth quintiles. Table 1 shows the wealth and educational disparities in access to PHC services in the four studied contexts using the three disparity measures.

**Table 1:** Education and wealth disparities in access to PHC services in Cameroon, DRC, Mali, and Nigeria

Type of disparity	Indicator	Country	Absolute difference		Concentration Index			Regression Coefficients	
			Least^**a**^	Highes^**b**^	***P***-value^**c**^	CI	P value^**d**^	OR^**e**^	P value^**f**^
Education	Geographic Barriers	Cameroon	57.42	30.37	0.001	−0.25	< 0.001	0.72	0.006
		DRC	48.81	31.32	< 0.001	−0.16	< 0.001	0.86	0.142
		Mali	33.75	14.6	< 0.001	−0.15	< 0.001	0.65	0.001
		Nigeria	35.84	18.45	< 0.001	−0.18	< 0.001	0.61	< 0.001
	Financial Barriers	Cameroon	81.44	58.52	< 0.001	− 0.23	< 0.001	0.57	< 0.001
		DRC	79.93	59.85	< 0.001	−0.19	< 0.001	0.56	< 0.001
		Mali	46.37	24.34	< 0.001	−0.17	< 0.001	0.55	< 0.001
		Nigeria	55.9	37.62	0.875	−0.21	< 0.001	0.51	< 0.001
Wealth	Geographic Barriers	Cameroon	62.7	23.41	< 0.001	−0.32	< 0.001	0.37	< 0.001
		DRC	53.09	20.71	< 0.001	−0.27	< 0.001	0.60	0.002
		Mali	45.18	14.14	< 0.001	−0.21	< 0.001	0.23	< 0.001
		Nigeria	45.52	12.78	< 0.001	−0.25	< 0.001	0.35	< 0.001
	Financial Barriers	Cameroon	84.11	49.45	0.127	−0.28	< 0.001	0.18	< 0.001
		DRC	80.83	50.09	0.04	−0.25	< 0.001	0.27	< 0.001
		Mali	54.94	25.84	0.003	−0.24	< 0.001	0.12	< 0.001
		Nigeria	61.26	28.16	< 0.001	−0.27	< 0.001	0.18	< 0.001

a: includes women with no education or in the first economic quintile, b: includes women with secondary or more education or in the 5th economic quintile, c: adjusted Wald test of equal proportions, b: t-test assuming unequal variance, c: model was adjusted for women age, employment, urban-rural status, and the number of children in the family, d: t-test of random intercept models.

### Disparities in geographic and financial barriers to care by conflict intensity

When neighborhoods were classified by the intensity of surrounding conflict events, those with medium or high-intensity conflict had more pronounced educational and wealth disparities in PHC access than those with no or low-intensity conflict in the four studied contexts. For instance, in DRC, those with no education living in neighborhoods surrounded by medium or high intensity conflict perceived 23% more geographic barriers and 30% more financial barriers to care than women with secondary or more education living in the same type of cluster. On the other hand, women with no education living in neighborhoods surrounded by no or low-intensity conflict perceived only 11% more geographic barriers and 12% more financial barriers than women with secondary or more education living in the same type of clusters.

Similarly, Congolese women in the first wealth quintile living in neighborhoods surrounded by medium or high intensity conflict perceived 35% more geographic barriers and 33% more financial barriers to PHC services than women in the fifth wealth quintile living in the same category of neighborhoods versus an absolute difference of 22 and 20% respectively among women living in clusters surrounded by no or low-intensity conflict. This pattern was also observed in Cameroon and Nigeria and in Mali, using the absolute difference as a measure of disparity.

**Fig. 3:**
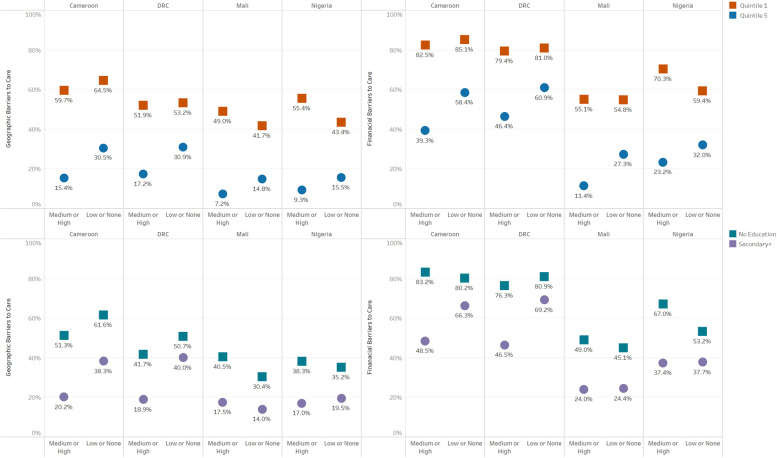
Perceived geographic & financial barriers to PHC access by conflict intensity among the most and the least privileged subgroups of educational status and wealth quintiles in the four studied contexts

It was also noted that despite a higher magnitude of disparity in neighborhoods surrounded by medium or high-intensity conflict compared to no or low intensity conflict, there were several instances where less privileged sub-groups in no or low intensity conflict in Cameroon and DRC perceived more geographic and financial barriers to care compared to their counterparts in medium or high-intensity conflict. Figure 3 shows the perceived barriers in access to PHC services by conflict intensity among the most and the least privileged sub-groups of educational status and wealth index.

Meanwhile, point estimates and concentration index z-tests suggest that the degree of educational and financial disparities is similar or greater in neighborhoods surrounded by medium or high intensity conflict than those with no or low-intensity conflict. The only exception to such observation was noted in Mali, where clusters with no or low-intensity conflict had higher educational disparities in financial access to PHC than clusters with medium or high conflict; however, the observed difference was small (Z = 2.03, *p* = 0.043). Table 2 shows the educational and wealth disparities in access to PHC services in the four studied contexts using the concentration index. Using random intercept models (mixed-effects logistic regression) in both types of neighborhoods, the odds of perceiving financial or geographic barriers were lower among women with secondary or more education or in the fifth wealth quintiles than women with no education or in the first wealth quintile, respectively.

**Table 2:** Education and wealth disparities in access to PHC services in Cameroon, DRC, Mali, and Nigeria by conflict intensity (using concentration index)

Type of disparity	Indicator	Country	Conflict intensity				Difference	
			Medium or high		low or none			
			CI	P-value	CI	P-value	Z test	P-value
Education	Geographic Barriers	Cameroon	−0.26	< 0.001	− 0.22	< 0.001	− 0.89	0.375
		DRC	−0.18	< 0.001	−0.09	< 0.001	−2.43	0.015
		Mali	−0.10	< 0.001	− 0.14	< 0.001	1.43	0.153
		Nigeria	−0.20	< 0.001	− 0.16	< 0.001	− 1.49	0.137
	Financial Barriers	Cameroon	−0.31	< 0.001	− 0.15	< 0.001	−5.18	< 0.001
		DRC	−0.24	< 0.001	− 0.10	< 0.001	−3.62	< 0.001
		Mali	−0.11	< 0.001	− 0.18	< 0.001	2.03	0.043
		Nigeria	−0.30	< 0.001	− 0.17	< 0.001	−5.57	< 0.001
Wealth	Geographic Barriers	Cameroon	−0.31	< 0.001	− 0.31	< 0.001	− 0.01	0.988
		DRC	−0.22	< 0.001	−0.16	< 0.001	−1.19	0.235
		Mali	−0.29	< 0.001	−0.21	< 0.001	−1.69	0.090
		Nigeria	−0.31	< 0.001	−0.21	< 0.001	−3.31	0.001
	Financial Barriers	Cameroon	−0.36	< 0.001	−0.22	< 0.001	−3.56	< 0.001
		DRC	−0.22	< 0.001	−0.12	< 0.001	−2.14	0.032
		Mali	−0.24	< 0.001	−0.21	< 0.001	− 0.57	0.566
		Nigeria	−0.39	< 0.001	−0.22	< 0.001	−6.20	< 0.001

However, no statistically significant interaction between conflict intensity and either wealth or educational disparities was noted in any of the studied contexts except while assessing educational disparities in Nigeria, where a statistically significant interaction between conflict intensity and both the geographic and financial access to PHC was observed, t = − 2.28, *p* = 0.023, and t = − 1.98, *p* = 0.048 respectively). Table 3 shows the educational and wealth disparities in access to PHC services in the four studied contexts using random intercept models.

**Table 3:** Educational and wealth disparities in access to PHC services in Cameroon, DRC, Mali, and Nigeria by conflict intensity (using mixed-effects logistic regression)

Type of disparity	Indicator	Country	Conflict intensity				Interaction	
			Medium or high		low or none			
			OR^**a**^	SE	OR^**a**^	SE	t test^**a**^	P-value
Education	Geographic Barriers	Cameroon	0.782	0.150	0.683**	0.100	0.22	0.828
		DRC	0.719	0.153	0.910	0.101	−1.12	0.263
		Mali	0.925	0.212	0.584***	0.093	1.59	0.112
		Nigeria	0.500***	0.054	0.680***	0.053	−2.28	0.023
	Financial Barriers	Cameroon	0.459***	0.086	0.637**	0.103	−1.63	0.105
		DRC	0.512***	0.127	0.585***	0.090	−0.56	0.576
		Mali	0.713	0.144	0.506***	0.060	1.05	0.296
		Nigeria	0.441***	0.036	0.532***	0.039	−1.98	0.048
Wealth	Geographic Barriers	Cameroon	0.448**	0.127	0.356***	0.089	0.68	0.499
		DRC	0.607	0.187	0.644*	0.124	−0.31	0.760
		Mali	0.250**	0.112	0.232***	0.061	0.09	0.931
		Nigeria	0.297***	0.061	0.373***	0.047	−1.14	0.255
	Financial Barriers	Cameroon	0.242***	0.074	0.143***	0.041	1.21	0.229
		DRC	0.326***	0.095	0.280***	0.054	0.04	0.970
		Mali	0.098***	0.038	0.116***	0.028	−0.49	0.624
		Nigeria	0.169***	0.032	0.192***	0.021	−1.09	0.277

a: model was adjusted for women’s age, employment, urban-rural status, and the number of children in the family.

*** p < 0.001, ** *p* < 0.01, * *p* < 0.05

## Discussion

This study addresses a literature gap by documenting the effect of geographic proximity to different levels of conflict intensity on the educational and wealth disparities in access to PHC services within fragile states. Regardless of conflict intensity, both wealth and educational disparities were observed in access to PHC services in the four studied contexts, which aligns with previous studies in several Sub-Saharan African countries [35–40].

Akseer et al. [4] reported that access to essential maternal and reproductive health services was far worse among the less educated and the poorer in conflict versus non-conflict affected countries. Similarly, a pooled multi-country analysis by Bendavid et al. highlighted reduced access to maternal and child health services among the poorest and the less educated compared to the wealthier and the more educated in conflict and fragile situations [24]. However, previous studies did not investigate sub-national disparities within conflict-affected fragile states themselves. In this study, we found that both wealth and educational disparities in access to PHC services can be exacerbated by the geographic proximity to sub-national organized violence.

Using three different measures of disparities, we observed a higher magnitude of wealth than educational disparities, as well as a higher magnitude of financial than geographic access barriers in most of the studied contexts. This observation may be a reflection of the nature of health financing in conflict and fragile situations, with higher out of pocket payments, lower government expenditure on health, and external dependency [41]. PHC particularly receives lower funding in many low-income conflict and fragile situations with the reliance on user fees for facility operation [41]. The latter was specifically reported in DRC by Barroy et al .[42], who found that only a quarter of Congolese government health expenditure was allocated to facility operations, leaving facilities to depend on fees from service payment to cover operational costs..

We also noted that a higher PHC access score does not necessarily mean lower disparities in access to care. For example, despite finding a better access score in Congolese neighbourhoods with medium or high-intensity conflict compared to no or low-intensity conflict, statistically significant higher disparities were observed in medium or high intensity conflict. This is not the first time that conflict zones have shown better health outcomes than non-conflict affected areas in DRC. In a subnational analysis of maternal and child health service coverage in DRC, North Kivu consistently showed higher coverage compared to the national average from 2001 to 2013 [43]. However, our findings suggest that a breakdown of key health indicators among different social groups and wealth strata is important for a more accurate understanding of the situation among vulnerable groups.

This study has important programmatic implications. First, disparities are likely to exist within conflict-affected fragile states regardless of conflict intensity. Therefore, interventions should target less-advantaged groups in the entire country, not only areas where sub-national conflict is reported. The latter is specifically important with conflict-associated population displacement as health systems in relatively stable areas can be strained as populations move away from conflict-affected areas in search of safety, livelihoods, and essential services. Second, this study sheds light on the scenario where medium or high intensity conflict could have an overall better access score yet, there is unfair distribution among social groups, underscoring the need for governments, development partners and humanitarian organizations to prioritize and monitor the access of less advantaged social groups in these settings. Lastly, our study highlights the importance of disaggregation of health metrics at the sub-national level and among different social strata instead of reporting a single national measure that may obscure real bottlenecks, especially with proximity to medium or high intensity conflict.

To our knowledge, the is the first study that systematically investigates disparities in PHC access, including the potential effect of conflict intensity at the household cluster level. Four neighbouring countries were compared using standardized data sources and definitions so consistent patterns can be identified within countries. Although within each country, there are other important contextual factors besides conflict, the “within” country comparisons adds the additional benefit of comparing areas with similar governing bodies and health system structures that cannot always be achieved when comparing different countries. This study also shows the utility of population-based surveys in providing insights on the patterns of healthcare utilization in conflict and fragile situations; specifically unmasking disparities that may be hidden in national and province/state-level reporting of service coverage.

Meanwhile, several factors have limited the presented analysis. For example, the study could not account for the effect of population displacement as no reliable data was found for geographic linkage at the household cluster level. The latter may have affected the presented results due to the difference in the estimated denominators. Specifically, the calculation of conflict intensity at the household cluster level was based on the 2015 population estimates. Analysed values may not reflect the actual cluster size or the effect of population displacement when DHS data were collected. Additionally, this study measured access using the DHS question on perceived barriers to care, which may not be specific to PHC. However, the question did not ask about emergency care or even labor so it can be used as a proxy for PHC access. Also, access was only examined from the demand side; future studies should assess disparities in more upstream supply factors. Specifically, analysis of disparities using heath facility surveys can allow for a more comprehensive understanding of the impact of conflict on disparities in PHC access.

## Conclusion

The magnitude of both the educational and wealth disparities in access to PHC services was higher with proximity to medium or high-intensity conflict within Cameroon, DRC, Mali, and Nigeria. However, disparities are likely to exist in the entire country regardless of conflict intensity. Studies examining the effect of population displacement and upstream supply factors at the health facility level are needed for a more comprehensive understanding of the effect of conflict on healthcare access.

## Supplementary Information

**Additional file 1.**

## Abbreviations

PHC: Primary Healthcare
DRC: Democratic Republic of Congo
DHS: Demographic Health Survey
EA: Enumeration Area
PHCPI: Primary Health Care Performance Initiative
SDGs: Sustainable Development Goals
UCDP: Uppsala Conflict Data Program
WHO: World Health Organization
