# Supplementary material for: Access to primary healthcare Services in Conflict-Affected Fragile States: a subnational descriptive analysis of educational and wealth disparities in Cameroon, Democratic Republic of Congo, Mali, and Nigeria
Source: Int J Equity Health. 2021 Dec 11;20:253. doi: 10.1186/s12939-021-01595-z (PMC8665620; doi:10.1186/s12939-021-01595-z)
Supplement: Supplementary file 1 — Additional file 1. [file 12939_2021_1595_MOESM1_ESM.docx]

**Table ‎S‑1: Characteristics of demographic health surveys included in the analysis.**

|  | **Cameroon** | **DRC** | **Mali** | **Nigeria** |
| --- | --- | --- | --- | --- |
| **Type of Survey** | **DHS-VII** | **DHS-VI** | **DHS-VII** | **DHS-VII** |
| **Type of sample** | Two-stage  stratified sample | Three-stage  stratified sample* | Two-stage  stratified sample | Two-stage  stratified sample |
| **Total number of clusters** | 470 | 540 | 379 | 1400 |
| **Households per cluster** | 28 | 34 | 26 ** | 30 |
| **Households(n)** | 11,710 | 18,171 | 9,510 | 40,427 |
| **Interviewed**  **Females (15-49) (n)** | 13,527 | 18,827 | 10,519 | 41,821 |
| **Fieldwork duration** | 06/2018-  12/2018 | 08/2013-  02/2014 | 08/2018-  11/2018 | 08/2018-  12/2018 |
| **Field restrictions** | South-West | ------- | Kidal*** | Borno |

* Two-stage stratified sample in statutory towns and cities of established provinces, three-stage stratified sample in rest of established provinces and new provinces

** 35 households per cluster was selected in Kidal, Gao, Tombouctou

*** Insecurity limited data collection in rural areas; only urban areas were visited

**Table ‎S‑2: Equations for multilevel logistic regression models**

|  | Educational disparities | Economic disparities |
| --- | --- | --- |
| Simple model (fit separately for each category of conflict intensity) | 𝑙𝑜𝑔𝑖𝑡(𝜋𝑖𝑗) = 𝛽0 + 𝛽1𝑎𝑔𝑒𝑖𝑗 + 𝛽2 1.edu𝑖𝑗 + **𝛽3 2.edu𝑖𝑗** + 𝛽4.employ𝑖𝑗+ 𝛽5 𝑐ℎ𝑖𝑙𝑑𝑟𝑒𝑛𝑖𝑗 + 𝛽6 urban𝑖𝑗 +u𝑖𝑗 | 𝑙𝑜𝑔𝑖𝑡(𝜋𝑖𝑗) = 𝛽0 + 𝛽1𝑎𝑔𝑒𝑖𝑗 + 𝛽2 1.quintile𝑗 + 𝛽3 2.quintile𝑖𝑗 + 𝛽4 3.quintile𝑖𝑗+ **𝛽5 4.quintile𝑖𝑗** +𝛽6 employ𝑖𝑗+ 𝛽7 𝑐ℎ𝑖𝑙𝑑𝑟𝑒𝑛𝑖𝑗 + 𝛽8 urban𝑖𝑗+ +u𝑖𝑗 |
| Interaction model | 𝑙𝑜𝑔𝑖𝑡(𝜋𝑖𝑗) = 𝛽0 + 𝛽1𝑎𝑔𝑒𝑖𝑗 + 𝛽2 1.edu𝑖𝑗 + 𝛽3 2.edu𝑖𝑗 + 𝛽4.employ𝑖𝑗+ 𝛽5 𝑐ℎ𝑖𝑙𝑑𝑟𝑒𝑛𝑖𝑗 + 𝛽6 urban𝑖𝑗+ 𝛽7 conflict𝑖𝑗 +𝛽8.edu𝑖𝑗 # conflict+ **𝛽9 2.edu𝑖𝑗 #conflict**+u𝑖𝑗 | 𝑙𝑜𝑔𝑖𝑡(𝜋𝑖𝑗) = 𝛽0 + 𝛽1𝑎𝑔𝑒𝑖𝑗 + 𝛽2 1.quintile𝑗 + 𝛽3 2.quintile𝑖𝑗 + 𝛽4 3.quintile𝑖𝑗+ 𝛽5 4.quintile𝑖𝑗 +𝛽6 employ𝑖𝑗+ 𝛽7 𝑐ℎ𝑖𝑙𝑑𝑟𝑒𝑛𝑖𝑗 + 𝛽8 urban𝑖𝑗+ 𝛽9 conflict𝑖𝑗+ 𝛽10 1.quintile𝑗 # conflict+ 𝛽11 2.quintile𝑖𝑗#conflict + 𝛽12 3.quintile𝑖𝑗#conflict+ **𝛽13 4.quintile𝑖𝑗 #conflict**+u𝑖𝑗 |

Coefficients and statistics in bold were reported

| Sociodemographic characteristic | Sub-group | Cameroon | | | DRC | | | Mali | | | Nigeria | | | |
| --- | --- | --- | --- | --- | --- | --- | --- | --- | --- | --- | --- | --- | --- | --- |
|  |  | **Medium or High** | **Low or none** | **Total** | **Medium or High** | **Low or none** | **Total** | **Medium or High** | **Low or none** | **Total** | **Medium or High** | **Low or none** | **Total** |  |
|  |  | % | % | no | % | % | no | % | % | no | % | % | no |  |
| Economic quintile | Poorest | 31.88 | 68.12 | 2,001 | 8.29 | 91.71 | 4,366 | 40.63 | 59.37 | 1,868 | 21.2 | 78.8 | 7,595 |  |
|  | Poorer | 32.97 | 67.03 | 2,881 | 7.97 | 92.03 | 3,740 | 42.03 | 57.97 | 1,837 | 32.17 | 67.83 | 8,257 |  |
|  | Middle | 31.07 | 68.93 | 3,479 | 12.67 | 87.33 | 3,655 | 36.66 | 63.34 | 1,961 | 36.55 | 63.45 | 8,737 |  |
|  | Richer | 38.00 | 62.00 | 3,158 | 22.86 | 77.14 | 3,390 | 27.56 | 72.44 | 2,344 | 40.1 | 59.9 | 8,760 |  |
|  | Richest | 43.41 | 56.59 | 3,158 | 69.37 | 30.63 | 3,676 | 11.24 | 88.76 | 2,509 | 40.36 | 59.64 | 8,002 |  |
| Highest educational level | None | 33.9 | 66.1 | 2,767 | 17.55 | 82.45 | 3,357 | 34.19 | 65.81 | 6,970 | 21.04 | 78.96 | 14,062 |  |
|  | Primary | 32.11 | 67.89 | 4,301 | 14.4 | 85.6 | 7,320 | 25.02 | 74.98 | 1,371 | 40.97 | 59.03 | 6,334 |  |
|  | Secondary | 37.45 | 62.55 | 6,615 | 31.53 | 68.47 | 7,589 | 21.63 | 78.37 | 1,974 | 40.41 | 59.59 | 16,645 |  |
|  | Higher | 44.67 | 55.33 | 994 | 73.44 | 26.56 | 561 | 12.25 | 87.75 | 204 | 44.57 | 55.43 | 4,310 |  |
| Employment Status | Unemployed | 32.90 | 67.10 | 5583 | 29.26 | 70.74 | 5984 | 30.71 | 69.29 | 5493 | 28.53 | 71.47 | 14,538 |  |
|  | Employed | 37.42 | 62.58 | 9094 | 21.01 | 78.99 | 12830 | 29.67 | 70.33 | 5026 | 37.50 | 62.50 | 26,813 |  |
| Age of women (years) | Mean | 30.37 | 29.53 | 29.88 | 27.54 | 28.33 | 28.09 | 29.26 | 28.17 | 28.48 | 29.34 | 28.94 | 29.08 |  |
|  | Sd | 11.86 | 11.48 | 11.65 | 9.13 | 9.31 | 9.27 | 9.10 | 9.04 | 9.07 | 9.57 | 9.64 | 9.62 |  |
| Children per woman (number) | Mean | 2.28 | 2.31 | 2.30 | 2.30 | 2.79 | 2.65 | 3.07 | 2.84 | 2.91 | 2.35 | 2.70 | 2.58 |  |
|  | Sd | 2.34 | 2.32 | 2.33 | 2.51 | 2.51 | 2.52 | 2.45 | 2.44 | 2.44 | 2.29 | 2.54 | 2.46 |  |
| Total (women) | | 35.7 | 64.3 | 14,677 | 23.63 | 76.37 | 18,827 | 30.21 | 69.79 | 10,519 | 34.34 | 65.65 | 41,351 | |

**Table S-3: Sociodemographic characteristics of women included in the analysis by conflict intensity in Cameroon, DRC, Mali, and Nigeria**
